# Direct cardiac versus systemic effects of inorganic nitrite on human left ventricular function

**DOI:** 10.1152/ajpheart.00081.2021

**Published:** 2021-05-21

**Authors:** Kevin O’Gallagher, Ana R. Cabaco, Matthew Ryan, Ali Roomi, Haotian Gu, Luke Dancy, Narbeh Melikian, Philip J. Chowienczyk, Andrew J. Webb, Ajay M. Shah

**Affiliations:** ^1^Department of Cardiology, School of Cardiovascular Medicine & Sciences, King’s College London British Heart Foundation Centre of Research Excellence, London, United Kingdom; ^2^Department of Clinical Pharmacology, School of Cardiovascular Medicine & Sciences, King’s College London British Heart Foundation Centre of Research Excellence, London, United Kingdom

**Keywords:** diastolic function, HFpEF, inorganic nitrite, nitric oxide, pressure-volume relationship

## Abstract

Inorganic nitrite is a source of nitric oxide (NO) and is considered as a potential therapy in settings where endogenous NO bioactivity is reduced and left ventricular (LV) function impaired. However, the effects of nitrite on human cardiac contractile function, and the extent to which these are direct or indirect, are unclear. We studied 40 patients undergoing diagnostic cardiac catheterization who had normal LV systolic function and were not found to have obstructive coronary disease. They received either an intracoronary sodium nitrite infusion (8.7–26 µmol/min, *n* = 20) or an intravenous sodium nitrite infusion (50 µg/kg/min, *n* = 20). LV pressure-volume relations were recorded. The primary end point was LV end-diastolic pressure (LVEDP). Secondary end points included indices of LV systolic and diastolic function. Intracoronary nitrite infusion induced a significant reduction in LVEDP, LV end-diastolic pressure-volume relationship (EDPVR), and the time to LV end-systole (LVEST) but had no significant effect on LV systolic function or systemic hemodynamics. Intravenous nitrite infusion induced greater effects, with significant decreases in LVEDP, EDPVR, LVEST, LV dP/d*t*_min_, tau, and mean arterial pressure. Inorganic nitrite has modest direct effects on human LV diastolic function, independent of LV loading conditions and without affecting LV systolic properties. However, the systemic administration of nitrite has larger effects on LV diastolic function, which are related to reduction in both preload and afterload. These contractile effects of inorganic nitrite may indicate a favorable profile for conditions characterized by LV diastolic dysfunction.

**NEW & NOTEWORTHY** This is the first study to assess the direct and indirect effects of inorganic nitrite on invasive measures of left ventricular function in humans in vivo. Inorganic nitrite has a modest direct myocardial effect, improving diastolic function. Systemic administration of nitrite has larger effects related to alterations in cardiac preload and afterload. The changes induced by nitrite appear favorable for potential use in conditions characterized by LV diastolic dysfunction.

## INTRODUCTION

Nitric oxide (NO) has important roles in the physiological regulation of cardiovascular function, whereas dysfunction of endogenous NO production or NO-cyclic GMP (cGMP) signaling are implicated in the pathophysiology of several cardiovascular diseases (1, 2). Accordingly, strategies to increase local tissue concentrations of NO or to enhance NO-dependent signaling may have therapeutic potential. Inorganic nitrite (NO_2_^−^) is of interest in this regard as it can be reduced to NO and have effects similar to NO donors but tolerance does not develop to its effects with continued use, unlike the case with organic nitrates (3, 4). Inorganic nitrite is a vasodilator, affecting both arterial (5–7) and venous tone (6). In the coronary bed, nitrite is relatively selective for conduit versus resistance vessels (8). When given via intravenous infusion, nitrite causes vasodilatation and a reduction in central blood pressure (7). Nitrite also inhibits platelet aggregation (9, 10) and can improve mitochondrial efficiency (11). Previous experimental and clinical studies have therefore explored the potential therapeutic benefit of nitrite in conditions such as myocardial ischemia-reperfusion (12, 13), pulmonary hypertension (14), cerebral vasospasm (15), and impaired exercise capacity in heart failure (16, 17).

Endogenously generated NO induces direct acute effects on the onset of myocardial relaxation and on diastolic distensibility, independent of changes in systolic function or systemic loading. A selective NO- and cGMP/protein kinase G (PKG)-dependent earlier onset of relaxation without change in systolic function was found in isolated mammalian cardiomyocytes and isolated hearts (18, 19). NO also reduced diastolic stiffness (19). Similar effects on onset of LV relaxation and LV diastolic distensibility were observed in human subjects in vivo after acute intracoronary infusion of substance P to trigger the endogenous release of NO (20). Consistent with these effects, it has been suggested that dysfunction of NO-cGMP signaling contributes to left ventricular (LV) diastolic dysfunction both experimentally and in patients (21–23). As such, the clinical utility of nitrite to enhance relaxation and diastolic function is of interest. However, the direct myocardial effects of nitrite in the human heart and their relationship to its systemic effects have not been established. In this study, we investigated the effect of either intracoronary or intravenous nitrite infusion on LV contractile function.

## METHODS

### Participants

Invasive LV pressure-volume (PV) studies were performed on patients (*n* = 40) with suspected coronary artery disease who were referred for diagnostic coronary angiography. All subjects were known to have normal left ventricular systolic function on echocardiography. Written informed consent was obtained before cardiac catheterization, and the research study was performed at the end of the diagnostic procedure if there was an absence of significant epicardial coronary artery disease (<50% stenosis on coronary angiography and/or a fractional flow reserve >0.80). Patients were also excluded if they had heart failure (either a preexisting diagnosis or a current clinical syndrome consistent with heart failure), clinically significant valve disease, or had a history of glucose-6-phosphate dehydrogenase deficiency. Patients were required to be in sinus rhythm at the time of assessment, with atrial fibrillation and ventricular bigeminy/trigeminy being considered excluding factors. The study complied with the *Declaration of Helsinki* and was approved by the Institutional Ethics Committee (Reference: 12/LO/1067).

### Study Protocols

We studied either intracoronary nitrite infusion (*n* = 20) or intravenous nitrite infusion (*n* = 20). Radial or femoral arterial access was used for diagnostic coronary angiography at the discretion of the operator. A second arterial puncture was required for patients receiving intracoronary infusion. All patients received unfractionated heparin (5,000 IU bolus), with additional doses as required to maintain an activated clotting time (ACT) of >250 s.

For intracoronary infusion studies, a 6 Fr guide catheter was positioned at the ostium of the left main coronary artery. Patients first received a normal saline infusion for at least 5 min during which stable LV function parameters and BP were confirmed. They next received an intracoronary infusion of sodium nitrite (NaNO_2_, Tayside NHS, UK) at 8.7 µmol/min for 5 min, followed by 26 µmol/min for 5 min. The higher dose is estimated to achieve a maximal intracoronary concentration of ∼1,000 µM, using average resting coronary blood flow estimates, as described previously (8) and is equivalent to concentrations that when administered intraarterially in the peripheral circulation are locally active (i.e., devoid of systemic effects) (7). For intravenous infusion studies, sodium nitrite was administered at 50 µg/kg/min for 7 min via a canula in a large antecubital fossa vein. This dose was chosen to achieve physiologically significant reduction in systemic blood pressure and pulmonary capillary wedge pressure (i.e., both afterload and preload) (16). The local concentration of nitrite in the coronary circulation after systemic infusion is estimated to be >100-fold lower than after intracoronary infusion but achieves significant reduction in peripheral loading due to its generalized systemic actions. The direct myocardial actions of intracoronary nitrite could therefore be compared with the indirect effects (due to altered peripheral loading) of systemic nitrite. A micromanometer-conductance catheter (CD Leycom, The Netherlands) was placed in the left ventricle to record steady-state LV PV relations via an Intra-Cardiac Analyser (INCA) console (CD Leycom, The Netherlands). Recordings of PV relations were made immediately before and immediately after the nitrite infusion. Measurements were also made of heart rate, blood pressure and the ECG. All patients had a three-dimensional (3-D) transthoracic echocardiogram to estimate LV volumes, which were used for volumetric calibration. Dedicated software (CD Leycom, The Netherlands) was used for analysis of PV loop data including LV systolic and diastolic indices, and ventricular-arterial coupling (VA coupling, calculated as the ratio of arterial elastance to end-systolic elastance, Ea/Ees) (24, 25). Recordings were made at end-expiration. Ten beats were averaged to provide each data point.

We also quantified first-phase ejection fraction (EF1), which represents the proportion of blood ejected from the LV from the onset of systole to the time of the first peak of LV pressure. EF1 has been suggested as an index that assesses systolic function early during contraction and reflects systolic-diastolic coupling (26, 27).

### Sample Size and Study End Points

Previous work reported that a bicoronary infusion of sodium nitroprusside induced a decrease in LV end-diastolic pressure (LVEDP) from 18 ± 5 to 12 ± 3 mmHg (28), equating to an effect size of 1.37. We estimated that a single left coronary infusion of sodium nitrite might have an effect of two thirds of this magnitude, i.e., an effect size of 0.91. Therefore, with an α of 0.05 and power (1-β) of 0.95, the required sample size was 18 for a primary end-point of reduction in LVEDP. To allow an ∼10% margin for patients who may fail to complete the protocol (e.g., due to technical issues), 20 patients per group were recruited. Exploratory secondary end points included other measures of LV systolic and diastolic function.

### Statistical Analyses

Analyses were performed using GraphPad Prism 8 (GraphPad Software Inc). The Shapiro-Wilk test was used to assess normality. Data are expressed as means ± SD for parametric data and median [IQR] for nonparametric data; *n*, number of participants. Intracoronary data were compared by repeated measures of ANOVA with Tukey’s posttest for multiple comparisons (or nonparametric equivalent). Student’s *t* test was used to compare the intravenous data and the effect of intracoronary versus intravenous nitrite on PV parameters (change from baseline). Linear regression analysis was used to test for correlation between measures of LV structure and changes in the primary end point. *P* < 0.05 was considered statistically significant.

## RESULTS

The baseline characteristics of the patients included in the study are shown in Table 1. Risk factors for coronary artery disease such as hypertension, smoking, hypercholesterolemia, and diabetes were common but well matched between the intracoronary and intravenous infusion groups. All patients had a normal LV ejection fraction (EF) on echocardiography. However, some of the patients had an elevated LV mass index (LVMI) and left atrial volume index. The studies were performed without clinical complications in any patient.

**Table 1.** Baseline characteristics of study participants

Characteristic	Intracoronary Studies	Intravenous Studies	*P* Value
Female, *n* (%)	8 (40)	7 (35)	>0.99
Age, yr	64.8 ± 2.7	60.0 ± 2.3	0.18
BMI, kg/m^2^	30.8 ± 1.3	29.8 ± 1.0	0.54
Hypertension, *n* (%)	16 (80)	16 (80)	>0.99
Smoking history, *n* (%)	7 (35)	10 (50)	0.98
Hypercholesterolemia *n* (%)	12 (60)	9 (45)	0.82
Diabetes, *n* (%)	4 (20)	4 (20)	>0.99
Number of antihypertensives	1.8 ± 0.3	1.7 ± 0.3	0.81
Hemoglobin, g/L	135.0 [126.3, 142.8]	135.5 [124.3, 145.3]	0.62
Creatinine, mmol/L	75.5 [64.8, 89.0]	82.5 [74.0, 93.0]	0.29
Left ventricular ejection fraction (%)	58 [55.3, 62.2]	59.7 [56.05, 61.0]	0.98
LVESVI, mL/m^2^	19.8 [16.5, 24.1]	21.9 [15.4, 26.8]	0.55
LVEDVI, mL/m^2^	50.3 ± 3.3	51.0 ± 3.7	0.89
LV mass index, g/m^2^	84.3 ± 20.7	63.2 ± 19.9	0.47
LA volume index, mm^3^/m^2^	30.0 ± 9.1	26.3 ± 8.3	0.77
TAPSE, mm	2.3 ± 0.3	2.2 ± 0.3	0.81
E/e′_ave_	9.5 ± 3.6	8.5 ± 2.7	0.83

Parametric data are means ± SE, nonparametric data as median [IQR]. BMI, body mass index; LVESVI, LV end-systolic volume index; LVEDVI, LV end-diastolic volume index; TAPSE, tricuspid annular plane systolic excursion. Continuous variables analyzed by *t* test or nonparametric equivalent. Categorical variables analyzed by Kolmogorov–Smirnov text.

### Effects of Intracoronary Nitrite Infusion

**Figure 1.**
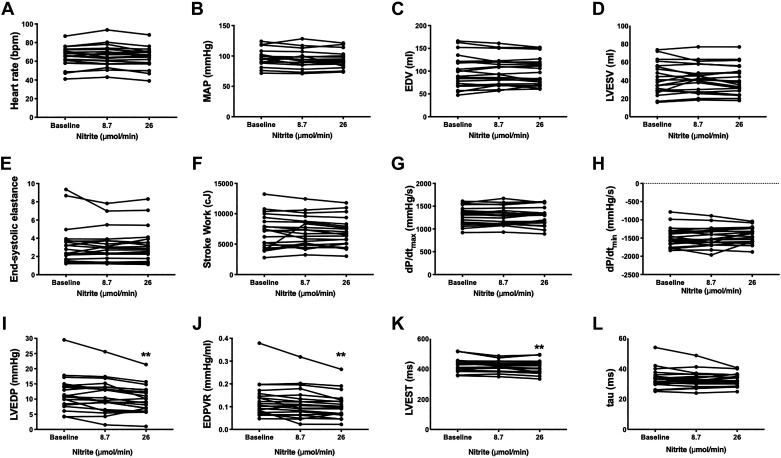
Effect of intracoronary nitrite on parameters of left ventricular (LV) function. *A*: heart rate; *B*: MAP, mean arterial pressure; *C*: LVEDV, LV end-diastolic volume; *D*: LVESV, LV end-systolic volume; *E*: Ees, end-systolic elastance; *F*: SW, stroke work; *G*: dP/d*t*_max_; *H*: dP/d*t*_min_; *I*: LVEDP, LV end-diastolic pressure; *J*: EDPVR, end-diastolic pressure-volume relation; *K*: LVEST, time to LV end-systole; *L*: tau. **P < 0.01 by one-way ANOVA with repeated measures and Tukey’s posttest. *n* = 17 for LVEST, *n* = 19 for MAP, *n* = 20 for all other parameters.

Intracoronary nitrite had no significant effect on heart rate or mean arterial blood pressure (MAP), consistent with a lack of systemic effect (Fig. 1, *A* and *B*). Markers of LV systolic function, namely LV end-systolic elastance (Ees), stroke work, and dP/d*t*_max_ were unaltered by intracoronary nitrite (Fig. 1, *E*–*G*). However, there was a significant decrease in the primary end point, LVEDP, following intracoronary nitrite (*P* = 0.004) (Fig. 1*I*). When considered as change from baseline, the 26 µmol/min nitrite dose decreased LVEDP by 1.9 mmHg [−3.3, −0.5] (mean [95% CI]), *P* = 0.006. Intracoronary nitrite also significantly decreased EDPVR, whereas the time to LV end-systole (LVEST) was decreased by 11 ms [−19, −4] (*P* = 0.002) at the higher dose of nitrite (Fig. 1, *J* and *K*). There were no significant changes in dP/d*t*_min_, tau, or LV volumes (Fig. 1, *C*–*D*, *H*, *L*). There was no significant change in VA coupling (Ea/Ees 0.6 ± 0.2 at baseline, 0.6 ± 0.2 following 26 µmol/min nitrite).

### Effects of Intravenous Nitrite Infusion

**Figure 2.**
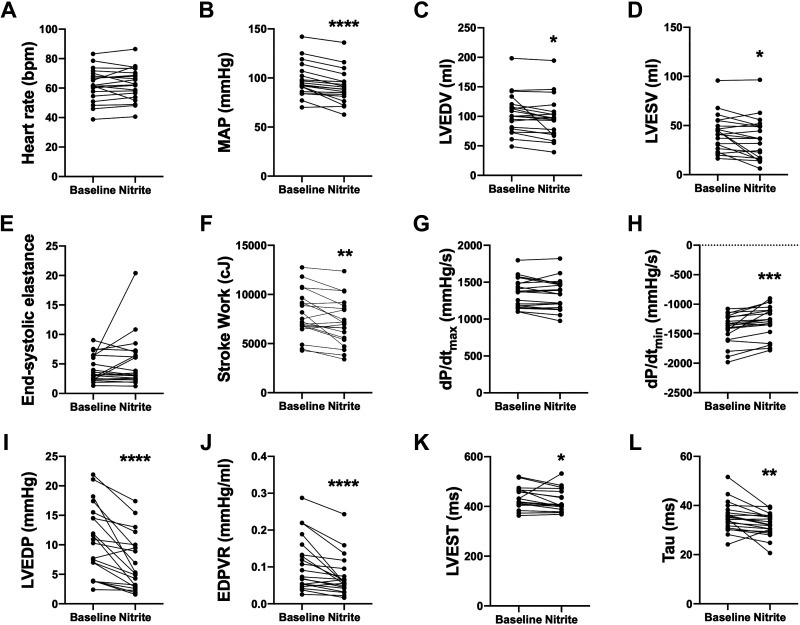
Effect of intravenous nitrite on parameters of left ventricular (LV) function. *A*: heart rate; *B*: MAP, mean arterial pressure; *C*: LVEDV, end-diastolic volume; *D*: LVESV, end-systolic volume; *E*: Ees, end-systolic elastance; *F*: SW, stroke work; *G*: dP/d*t*_max_; *H*: dP/d*t*_min_; *I*: LVEDP, LV end-diastolic pressure; *J*: EDPVR, end-diastolic pressure-volume relation; *K*: LVEST, time to LV end-systole; *L*: tau. *P < 0.05, **P < 0.01, ***P < 0.001, ****P < 0.0001 by Student’s *t* test. *n* = 19 for MAP and LVEST, *n* = 20 for all other parameters.

Intravenous nitrite resulted in a significant decrease in MAP of 6.9 mmHg [−4.3, −9.5] (mean [95% CI]), *P* < 0.001, but had no effect on heart rate (Fig. 2, *A* and *B*). Consistent with a reduction in afterload, the arterial elastance (Ea) decreased from 2.1 ± 0.7 to 1.9 ± 0.7 (*P* = 0.002). There was no change in the total peripheral resistance: mean change −0.7 [−2.2, +0.7] (mean [95% CI]), *P* = 0.3. Intravenous nitrite also induced a significant reduction in LVEDV {−8.3 mL [−15.4, −1.1] (mean [95% CI]), *P* = 0.03}, consistent with a decrease in preload (Fig. 2*C*). No changes were observed in Ees (regardless of whether the outlier data point is included or not, see Fig. 2*E*) or dP/d*t*_max_ while stroke work decreased significantly: −829 centijoules (cJ) [−1,327, −331] (mean [95% CI]), *P* = 0.003 (Fig. 2, *E*–*G*). Intravenous nitrite caused a significant reduction in LVEDP from a baseline of 10.6 mmHg [4.7, 15.3] (median [IQR]) to 5.2 mmHg [2.9, 9.9], *P* < 0.001 (Fig. 2*I*). Intravenous nitrite also resulted in significant decreases in EDPVR, LVEST, dP/d*t*_min_, and tau (Fig. 2, *H, J*–*L*). There was no significant change in ventricular-arterial coupling (Ea/Ees from 0.6 ± 0.2 to 0.5 ± 0.3, *P* = 0.06).

### Comparison of Intracoronary and Intravenous Nitrite

**Figure 3.**
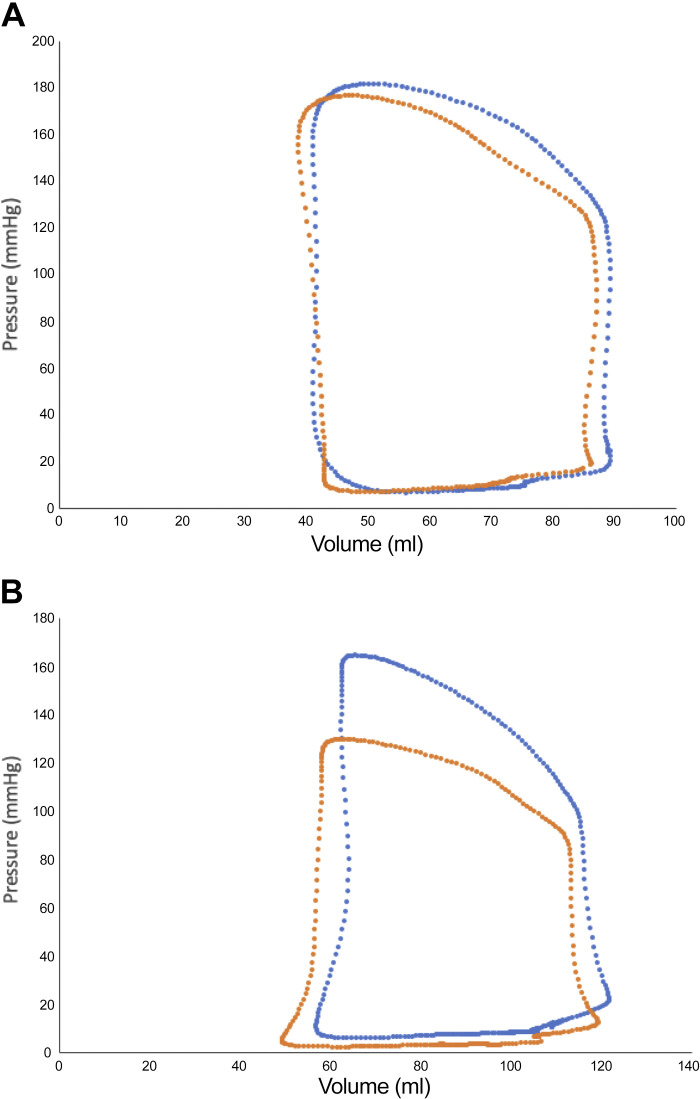
Representative pressure volume loops showing the effect of intracoronary nitrite (*A*) and intravenous nitrite (*B*). Blue loops represent baseline values and orange loops represent response to inorganic nitrite.

**Figure 4.**
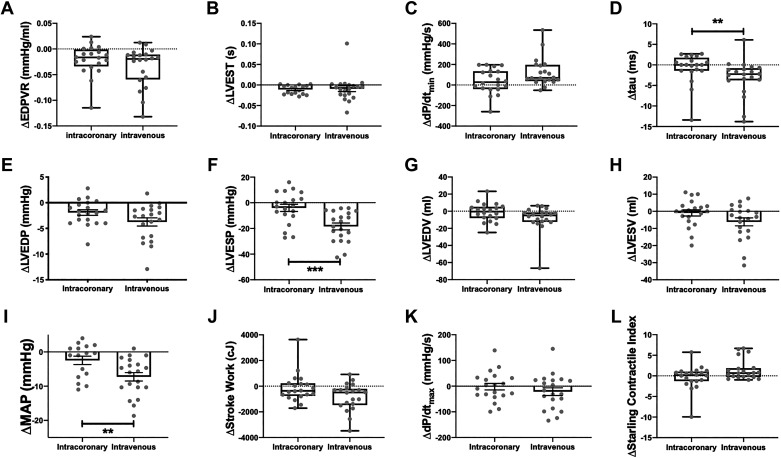
Comparison of effect between intracoronary and intravenous nitrite. *A*: EDPVR, end-diastolic pressure-volume relationship; *B*: LVEST, left ventricular electrosystolic time; *C*: dP/d*t*_min_; *D*: tau; *E*: LVEDP, LV end-diastolic pressure; *F*: LVESP, LV end-systolic pressure; *G*: LVEDV, LV end-diastolic volume; *H*: LVESV, time to LV end-systole; *I*: MAP, mean arterial pressure; *J*: stroke work; *K*: dP/d*t*_max_; *L*: Starling Contractile Index. **P < 0.01, ***P < 0.001 by Student’s *t* test. *n* = 36 for LVEST and MAP, *n* = 40 for all other parameters.

Representative PV loops showing the effect of intracoronary and intravenous nitrite infusion are shown in Fig. 3 and suggest that intravenous infusion had a larger effect. Figure 4 shows a quantitative comparison of the effects of intravenous and intracoronary (26 µmol/min nitrite) infusion. There was no significant difference at baseline between the groups in MAP {99.0 mmHg [89.7, 110.3] (median [IQR]) vs. 93.0 mmHg [86.0, 107.0] for intracoronary vs. intravenous} or LVEDP (11.0 mmHg [8.1, 14.3] vs. 10.6 mmHg [4.7, 15.3] for intracoronary vs. intravenous). Intravenous nitrite had significantly greater effects than intracoronary nitrite on MAP (Fig. 4*I*), LV end-systolic pressure (LVESP) (Fig. 4*F*), and tau (Fig. 4*D*). Although the mean decrease in LVEDP following intravenous nitrite was numerically greater than after intracoronary infusion (Fig. 4*E*), this was not statistically significant.

**Figure 5.**
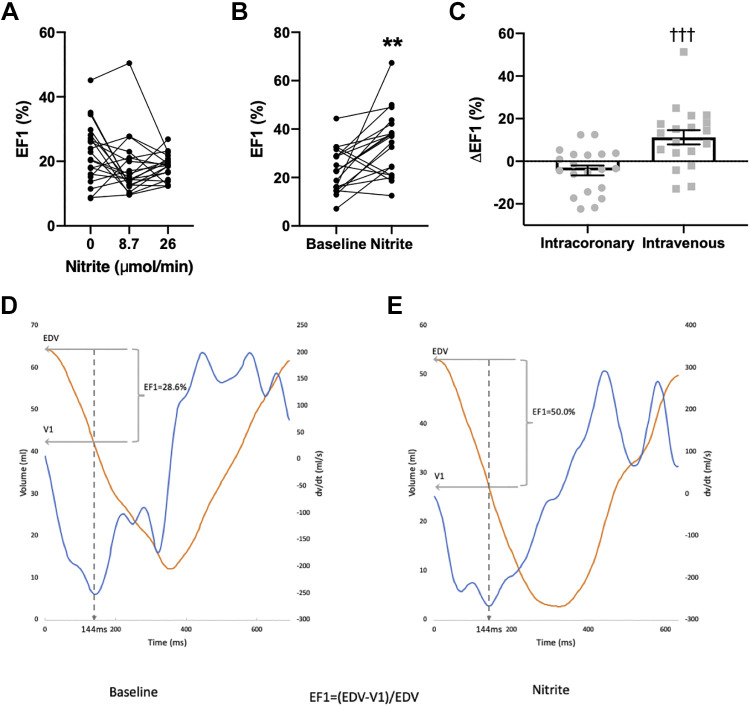
The effect of nitrite on EF1. *A*: intracoronary nitrite (*n* = 20). *B*: intravenous nitrite (*n* = 19). *C*: comparison of peak change after intracoronary vs. intravenous nitrite. *D*: representative baseline trace of left ventricular (LV) volume (orange) and dP/d*t*_max_ (blue) with EF1 calculation demonstrated. *E*: representative trace of LV volume (orange) and dP/d*t*_max_ (blue) following intravenous nitrite with EF1 calculation demonstrated. **P < 0.01 vs. baseline, †††*P* < 0.001 vs. intracoronary nitrite by Student’s *t* test.

Intracoronary nitrite had no significant effect on EF1 (*P* = 0.5 by one-way ANOVA) (Fig. 5*A*) but intravenous nitrite induced a marked increase in EF1 (Fig. 5, *B*–*C*). From a baseline of 23.0% ± 2.1%, the EF1 postnitrite was 34.2% ± 3.1%, a relative increase of ∼50% as illustrated by the representative traces in Fig. 5, *D* and *E*.

### Association between Baseline LV Structure and Function and the Effect of Nitrite

**Figure 6.**
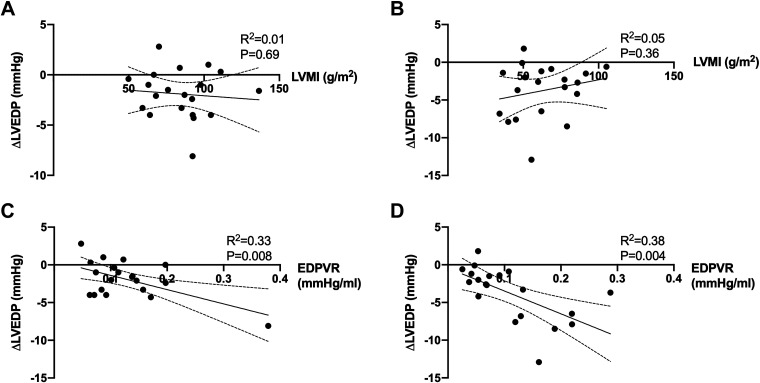
Correlation between left ventricular (LV) mass index (LVMI) and change in LV end-diastolic pressure (LVEDP) (*A* and *B*) and between baseline EDPVR and change in LVEDP (*C* and *D*). *A* and *C* show data for intracoronary nitrite and *B* and *D* show data for intravenous nitrite. *n* = 20 for all panels. Statistical analysis by logistical regression.

To assess whether interindividual variation in the response to nitrite might be related to baseline cardiac structure, we determined the association between LVMI and the magnitude of change in LVEDP but found no significant correlation either in the intracoronary or intravenous nitrite groups (Fig. 6, *A* and *B*). We also assessed whether the magnitude of reduction in LVEDP was related to baseline LV EDPVR. There was a significant association between the nitrite-induced decrease in LVEDP and baseline EDPVR for both the intracoronary group (*R*^2^ = 0.33, *P* = 0.008) (Fig. 6*C*) and the intravenous group (*R*^2^ = 0.38, *P* = 0.004) (Fig. 6*D*). It should be noted, however, that when the data are analyzed as percentage rather than absolute change in LVEDP, the association with baseline EDPVR is no longer significant in the intracoronary group but remains significant in the intravenous group.

## DISCUSSION

In this study, we have examined in detail that the direct and indirect acute effects of inorganic nitrite on contractile function of the human heart in patients undergoing diagnostic cardiac catheterization who did not have coronary disease or heart failure. We demonstrate several important findings that may have relevance to the potential therapeutic use of nitrite.

First, inorganic nitrite delivered via the intracoronary route induces a small but significant decrease in LVEDP and EDPVR and hastens the onset of LV relaxation (i.e., reduces LVEST). These effects are not accompanied by any change in blood pressure or heart rate, consistent with a local action on the heart. They occur without any alteration in indices of LV systolic function, indicating a selective effect on diastolic properties of the heart. No change in tau or LV dP/d*t*_min_ is observed following intracoronary nitrite, suggesting that while nitrite improves LV distensibility (a passive property), there is a lack of effect on active (ATP-dependent) myocardial relaxation. This pattern of effect on LV contractile function is entirely consistent with prior studies reporting similar direct myocardial effects of NO donors and NO-cGMP signaling on the onset of relaxation and diastolic stiffness, both in isolated preparations and in humans in vivo (18, 20, 28, 29). At a mechanistic level, such actions are considered to involve cGMP/protein kinase G (PKG)-mediated phosphorylation of troponin I and titin in cardiomyocytes (19, 30–32), although other NO-mediated mechanisms such as altered S-nitrosylation of proteins (33, 34) or an effect on sympathetic nerve activity (35, 36) may have a role. Taken together, the data from the intracoronary infusion study suggest that inorganic nitrite has a direct and selective action on the myocardium to reduce ventricular stiffness and hasten the onset of relaxation. However, the magnitude of these changes is small in patients with normal LV function.

Second, the results of the intravenous infusion studies indicate that systemic administration of nitrite significantly reduces LV preload (LVEDV) and afterload (MAP and Ea) and is associated with more marked effects on LV diastolic function than observed after intracoronary infusion. In addition to decreases in LVEDP, EDPVR, and LVEST, intravenous nitrite significantly accelerates tau and reduces LV dP/d*t*_min_. The effects of intravenous nitrite on LV function are likely to be due to the reduced loading of the heart rather than a direct myocardial action, as the local intracoronary concentration of nitrite achieved with systemic infusion is estimated to be substantially lower than with intracoronary infusion (see methods). Therefore, intravenous nitrite-induced effects on LV diastolic function appear to be driven mainly by its actions to reduce afterload and preload rather than a direct myocardial action in this patient group. The effect of nitrite to reduce afterload and preload is well established and the underlying mechanism is NO-mediated direct (endothelium-independent) vasodilation (5–7).

Third, intravenous nitrite infusion significantly increases EF1, a hemodynamic index that describes the proportion of LV ejection that occurs up to the time of maximal rate of ventricular contraction (26). EF1 has been proposed to reflect coupling between systolic and diastolic function and therefore to provide a more integrated readout of overall changes in cardiac function. EF1 may be reduced even in patients in whom the overall EF is within the normal range, in which group it strongly correlates with abnormal LV diastolic function as indexed by an elevated E/e′ ratio (indicating elevated filling pressures) on echocardiography (26). Furthermore, a reduced EF1 may predict a worse prognosis in patients with aortic stenosis or heart failure (27). In the present study, the intravenous nitrite-induced increases in EF1 suggest that an improvement in early ejection phase LV systolic function is induced in addition to the enhancement of diastolic function. Given that no change in EF1 was observed with intracoronary infusion, it is likely that the increase following intravenous nitrite infusion is due to the improved cardiac loading conditions rather than a direct myocardial effect. This is consistent with prior data that nitrite decreases pressure wave reflections in the arterial tree and decreases late systolic load on the LV (37) and that EF1 is highly sensitive to changes in late systolic load (26). The results with EF1 therefore further emphasize that the predominant effects of intravenous nitrite appear to relate to its actions to reduce afterload and preload.

A careful assessment of the systemic versus direct myocardial actions of either NO donors such as sodium nitroprusside or of inorganic nitrite on cardiac contractile function has not previously been undertaken. It was reported that intracoronary infusion of the NO donor sodium nitroprusside reduced LVEDP and LVEST (similar to the current study) in patients with normal LV function but that investigation did not involve measurement of PV relations nor the assessment of the effects of systemic administration (28). Recently, there has been considerable interest in the therapeutic potential of nitrite in conditions associated with decreased NO bioavailability, including heart failure (4). The current study was performed in patients with normal LV function to first establish the effects in this group but future studies need to also study its effects in patients with impaired LV function, where the magnitude or pattern of effect could be different. For example, the effects of the nitrite-NO pathway are reportedly greater under hypoxic or ischemic conditions (6). Extending the findings on the acute effects of nitrite to its potential therapeutic value also requires consideration of chronic administration. Nitrite can be administered in the form of oral nitrite salts (such as sodium nitrite) or plasma nitrite levels can be elevated via inorganic nitrate, for example, an oral nitrate salt or by ingestion of beetroot juice. Previous studies in which oral sodium nitrite (or nitrate) was administered chronically achieved postdose plasma nitrite concentration of a similar order of magnitude to those obtained after intravenous administration, whereas trough levels remain increased compared with placebo (38, 39). The chronic administration of beetroot juice is able to achieve slightly lower levels of plasma nitrite (40). Future studies to assess the effect of chronic elevation of plasma nitrite on cardiac function will be of value.

The pattern of effect of nitrite on LV contractile function raises the possibility that it could be of value in heart failure. Both Heart Failure with Reduced Ejection Fraction (HFrEF) and Heart Failure with Preserved Ejection Fraction (HFpEF) are characterized by significant LV diastolic dysfunction. In HFrEF, it is already well established that a reduction in the loading of the heart is beneficial and previous clinical trials in selected patient groups showed benefit from a combination of organic nitrates and hydralazine (41). HFpEF might theoretically be especially amenable to nitrite therapy, as there is quite good evidence of impaired NO/cGMP signaling and abnormal loading in this condition (42, 43). Furthermore, acute nitrite administration is reported to reduce pulmonary capillary wedge pressure (PCWP) during exercise in patients with HFpEF (16). However, a recent randomized trial of inhaled nitrite in HFpEF failed to show benefit with respect to echocardiographic filling pressures or exercise capacity (17), although inhaled nitrite may be considered more analogous to local than systemic delivery. A randomized trial studying the effects of 6-wk administration of an organic nitrate, isosorbide mononitrate, in patients with HFpEF also failed to show benefit (44). Whether chronic administration of inorganic nitrite (or dietary manipulation to elevate nitrite levels) has different effects in this patient group merits further study.

### Study Limitations

We studied a relatively small number of subjects, many of whom had risk factors for cardiovascular disease and were on medications. Some of the patients also had increased LV mass or raised LV filling pressures at baseline. As such, the study population is not comparable to healthy subjects. The possibility that the effects of nitrite may vary depending on risk factor cannot be excluded. The intracoronary group had a higher median LVMI than the intravenous group but the values were within the normal range in both groups. We looked for any correlation between LV mass and the magnitude of effect on LVEDP but found no significant relationship. The study design did not include a placebo group but all patients received an initial saline infusion (placebo) during which period the stability of LV function parameters was confirmed. It was not practical for logistic reasons to randomize allocation to intracoronary or intravenous nitrite, as the two procedures were technically different. Due to ethical and logistical considerations, it was also not feasible to perform both intracoronary and intravenous nitrite in the same patients as doing this would have made the study prohibitively long. The current study only looked at the acute effects of a single nitrite infusion at rest. It is possible that the effects may be larger upon exercise and the results also cannot necessarily be extrapolated to the effects of chronic administration. It is not known how much bioactive NO is released within the myocardium from a given dose of nitrite in this study and therefore the results are not generalizable to other NO donor drugs. This could be addressed in future studies by assessment of blood plasma sampled from both the coronary artery and the coronary sinus. EDPVR was assessed using the single beat method, rather than through vena cava occlusion to induce a loading change, but the single beat method is known to reliably detect the acute effects of interventions. As common in such clinical studies, our data set contained a number of outlier data points. However, repeating the analyses without inclusion of such outliers did not materially alter the overall pattern of results.

### Conclusion

We have undertaken a comprehensive characterization of the acute effects of intracoronary and intravenous nitrite on human cardiac contractile function, using doses designed to achieve solely local myocardial or solely peripheral vascular effects, respectively. Our findings demonstrate that nitrite has modest direct effects on LV diastolic distensibility and the onset of LV relaxation in subjects without heart failure. The effects of systemic infusion on diastolic function in this patient group are, however, much greater and involve altered cardiac loading. The overall profile of effect of nitrite may be beneficial in conditions characterized by LV diastolic dysfunction and merits further study.

## DATA AVAILABILITY STATEMENT

The data that support the findings of this study are available from the corresponding author upon reasonable request.

## GRANTS

This work was supported by a UK Medical Research Council Clinical Research Training Fellowship (MR/R017751/1 to K.’O.G.), the British Heart Foundation (RE/18/2/34213), and the Department of Health via a National Institute for Health Research (NIHR) Biomedical Research Centre award to Guy’s & St Thomas’ NHS Foundation Trust in partnership with King’s College London (IS-BRC-1215-20006) and King’s College Hospital NHS Foundation Trust.

## DISCLOSURES

The authors confirm that the PI for this paper is Prof. A. M. Shah and that he had direct clinical responsibility for patients. None of the other authors has any conflicts of interest, financial or otherwise, to disclose.

## AUTHOR CONTRIBUTIONS

K.’O.G., P.J.C., A.M.S. conceived and designed research; K.’O.G., A.R.C., M.R., A.R., L.D., N.M., and A.M.S. performed experiments; K.’O.G., A.R.C., M.R., H.G., and A.M.S. analyzed data; K.’O.G., P.J.C., A.J.W., and A.M.S. interpreted results of experiments; K.’O.G. prepared figures; K.’O.G. and A.M.S. drafted manuscript; K.’O.G., P.J.C., A.J.W., and A.M.S. edited and revised manuscript; K.’O.G., A.R.C., M.R., A.R., H.G., L.D., N.M., P.J.C., A.J.W., and A.M.S. approved final version of manuscript.
